# Gut microbiota metabolites in inflammatory bowel disease: advances in mechanistic insights

**DOI:** 10.3389/fimmu.2026.1827426

**Published:** 2026-06-10

**Authors:** Yuhong Liang, Yingchen Zhou, Pei Luo, Juanna Lin

**Affiliations:** 1 State Key Laboratory of Mechanism and Quality of Chinese Medicine & School of Pharmacy, Faculty of Medicine, Macau University of Science and Technology, Macau, China; 2 Department of Pharmacy, the First Affiliated Hospital of Medical College of Shantou University, Shantou, China

**Keywords:** gut microbiota metabolites, inflammatory bowel disease, intestinal mucosal immunity, mechanism of action, precision medicine

## Abstract

Inflammatory bowel disease (IBD), primarily comprising Crohn’s disease and ulcerative colitis, represents a group of chronic, relapsing intestinal inflammatory conditions mediated by immune dysregulation. Emerging evidence has established the gut microbiota and its metabolic products as central players in IBD pathogenesis. The human gut microbiota constitutes a vast and dynamic micro-ecosystem whose metabolic activities generate a diverse array of small molecules, including short-chain fatty acids, bile acids, and tryptophan-derived metabolites. These metabolites collectively form a “gut microbiota-metabolite-immune axis” that is deeply involved in maintaining intestinal homeostasis, and their dysregulation is closely linked to IBD initiation and progression. Patients with IBD typically exhibit significant alterations in gut microbial composition and function, with key metabolic perturbations characterized by reduced levels of SCFAs and secondary bile acids, as well as imbalances in specific amino acid-derived metabolites. SCFAs not only serve as essential energy substrates for colonic epithelial cells but also modulate immune responses and enhance barrier integrity through G protein-coupled receptors and inhibition of histone deacetylases. Bile acids contribute to barrier function and immune balance via activation of nuclear receptors such as the farnesoid X receptor and the G protein-coupled bile acid receptor 1. Tryptophan, metabolized by both host enzymes and the gut microbiota into kynurenine, serotonin, and various indole derivatives, participates in immunoregulation through pathways involving the aryl hydrocarbon receptor. These examples underscore the pivotal roles of gut microbial metabolites in both the pathogenesis and treatment of IBD. This review aims to synthesize recent advances in understanding the functions and molecular mechanisms of key gut microbial metabolites in IBD, with a focus on how they orchestrate the initiation and perpetuation of intestinal inflammation through complex immunoregulatory networks and modulate intestinal barrier function. By providing new insights into the mechanisms underlying IBD pathogenesis and intervention, this review seeks to establish a theoretical foundation for the development of novel diagnostic and therapeutic strategies targeting microbial metabolites.

## 1 Introduction

The human gut microbiota constitutes a highly complex, diverse, and dynamic micro-ecosystem, encompassing bacteria, fungi, viruses, archaea, and protozoa. Its collective genome exceeds the size of the human genome by more than 100-fold, representing a vast genetic repertoire adapted to the intestinal environment. This “meta-organism” performs numerous metabolic functions complementary to the host, such as polysaccharide fermentation and vitamin synthesis, thereby participating in various aspects of human physiology and linking closely to the pathogenesis of multiple diseases (1). Inflammatory bowel disease (IBD) is a heterogeneous, immune-mediated, and multifactorial inflammatory condition, primarily manifesting as two clinical entities: Crohn’s disease (CD) and ulcerative colitis (UC) (2). Characterized by a chronic relapsing and remitting course requiring lifelong management, IBD imposes substantial physical and psychological burdens on patients (3). Accumulating evidence indicates that IBD results from a complex interplay between dysregulated mucosal immune responses and impaired intestinal epithelial barrier function (4). Susceptibility and genetic polymorphisms associated with IBD are largely linked to the gut microbiota, and its alteration is considered a key trigger of chronic intestinal inflammation, although the precise underlying mechanisms remain incompletely defined (5).

Through their metabolic activities, gut microbes generate a vast array of metabolites via fermentation, including short-chain fatty acids (SCFAs), tryptophan (Trp) and indole derivatives, and bile acids (6). These small molecules can be synthesized *de novo* by the microbiota or derived from dietary or host-derived compounds (6). Microbial metabolites can enter the systemic circulation, achieving a broader distribution within the body than the microbes themselves. Advances in proteomics and metabolomics have revealed that these metabolites serve as crucial mediators of the gut’s regulatory influence on host immunity, exerting significant and diverse effects on both the pathogenesis and treatment of IBD. However, definitive causal relationships between specific metabolites and IBD pathogenesis remain to be fully elucidated (7). With a population of 1.4 billion, China’s accelerating urbanization and industrialization may further escalate the burden of IBD (8). In this context, precisely targeting and quantifying interventions based on gut microbial metabolites holds substantial clinical promise. This review aims to synthesize the potential roles of key gut microbial metabolites—specifically SCFAs, bile acids, and Trp metabolites—in the pathogenesis and treatment of IBD, thereby providing a theoretical foundation for the development of therapeutic strategies targeting the gut metabolome.

## 2 Dysregulation of gut microbiota metabolites in IBD

**Figure 1:**
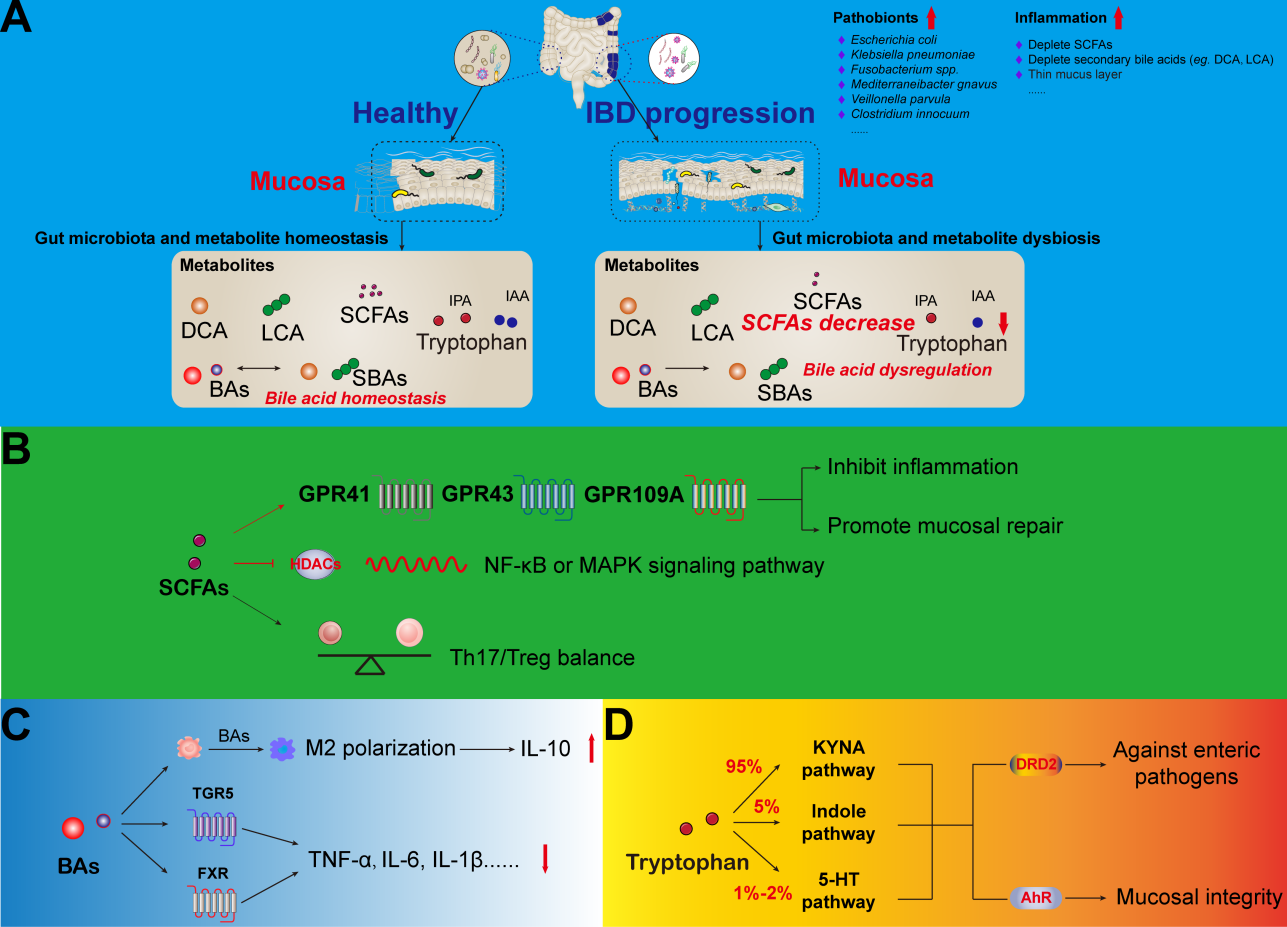
Schematic of main gut microbiota metabolites in IBD therapy research. **(A)** Comparison of gut microbiota metabolites in healthy and IBD progression-states. Healthy-state (Left): Normal gut microbiota with stable structure, balanced metabolites. IBD progression-states (Right): Microbial metabolite dysbiosis with overgrowth of pathogenic bacteria (e.g., *Escherichia coli*, *Klebsiella pneumoniae*), deplete SCFAs, disrupt BA regulation, reduce anti - inflammatory substances derived from tryptophan, and thin the mucus layer. **(B)** SCFAs in immune regulation and mucosal repair. SCFAs inhibit inflammation and promote mucosal repair via GPRs. They suppress HDACs to activate NF-κB or MAPK signaling, regulating Th17/Treg balance. **(C)** BAs in immune modulation and inflammation suppression. BAs activate TGR5 to promote M2 macrophage polarization and upregulate IL-10. Through FXR, they inhibit pro-inflammatory cytokines. **(D)** Tryptophan metabolism and intestinal barrier function. Tryptophan metabolizes via three pathways to maintain mucosal integrity.

Early evidence of metabolite alterations in IBD came from a seminal study by Franzosa et al. (9), who performed untargeted liquid chromatography-mass spectrometry (LC-MS) metabolomic profiling in an IBD patient cohort. They identified over 2,700 differentially abundant metabolites. Compared to non-IBD healthy controls, IBD patients exhibited elevated levels of primary bile acids, α-amino acids, and sphingolipids, alongside decreased levels of several metabolites, including cholesterol, benzodioxane, indoles, tetrapyrroles, and long-chain fatty acids. Notably, more than 50% of the detected metabolites were previously uncharacterized. A subsequent multi-omics study from the integrative Human Microbiome Project (iHMP) (10) revealed functional dysbiosis in the gut microbiota of IBD patients. This dysbiosis was characterized by an expansion of potentially pathogenic organisms, including adherent-invasive Escherichia coli, Klebsiella pneumoniae, Fusobacterium species, Staphylococcus mediterranei, Veillonella parvula, and Clostridium innocuum, coupled with a depletion of anaerobic short-chain fatty acid (SCFA)-producing bacteria. This compositional shift was associated with reduced metabolite diversity, increased primary bile acids, decreased secondary bile acids, depletion of vitamins B3 and B5 and SCFAs, and enrichment of acylcarnitines and polyunsaturated fatty acids. Intriguingly, another study identified increased levels of N-acylethanolamines in feces from both IBD patients and colitic mice. These compounds were shown to promote the growth of bacterial species that are typically increased in IBD while inhibiting those that are decreased, thereby actively shaping the dysbiotic microbial community (11) (**Figure 1A**).

## 3 Impact of gut microbiota metabolites on IBD pathogenesis

### 3.1 The dual role of SCFAs in IBD pathogenesis

While substantial evidence supports the protective role of SCFAs, particularly butyrate, in IBD pathogenesis, not all effective interventions for CD are associated with elevated SCFA levels. Exclusive enteral nutrition (EEN), a first-line therapy for pediatric CD, often correlates with reduced microbial diversity and decreased butyrate levels. However, strategies aimed at enriching butyrate-producing bacteria have not matched the clinical efficacy of EEN (12). A recent systematic review found insufficient evidence to support butyrate enemas for UC and noted a lack of robust data for CD (13). Oral butyrate formulations, including tablets and microcapsules, have shown some benefit in both UC and CD patients (14), suggesting potential as an adjunctive therapy, though further research is required.

In adaptive immunity, SCFAs exert dual modulatory effects on T-cell subsets. On one hand, butyrate and propionate promote the generation of anti-inflammatory regulatory T cells (Tregs) by inhibiting HDAC and upregulating Foxp3 expression, thereby contributing to immune homeostasis. On the other hand, butyrate can, at high concentrations, induce the differentiation of pro-inflammatory Th1 and Th17 effector T cells (15). In a dextran sulfate sodium (DSS)-induced colitis mouse model, physiologically relevant concentrations of butyrate primarily induced a regulatory T-cell phenotype, whereas high-dose butyrate significantly promoted an IFNγ-mediated pro-inflammatory response (16) accompanied by an expansion of Th1/Th17 subsets. Furthermore, butyrate (and to a lesser extent, propionate) can upregulate the expression of IFN-γ and granzyme B in CD8^+^ T cells (17), suggesting a potential role in activating cytotoxic T lymphocytes. Given that increased infiltration of CD8^+^ T cells producing perforin correlates with disease activity in IBD, these findings illustrate that SCFAs favor Treg-mediated anti-inflammatory responses under steady-state conditions but may exert pro-inflammatory effects in an inflammatory milieu, reflecting a dual regulatory function through concurrent activation of effector and regulatory T-cell pathways (15).

Regarding B-cell responses and humoral immunity, SCFAs promote B-cell differentiation into plasma cells and enhance antibody production by modulating gene expression (18). Specifically in mucosal immunity, butyrate, via GPR43, enhances microbiota-specific immunoglobulin A (IgA) responses in Peyer’s patches, thereby influencing local immune homeostasis (**Figure 1B**). These discoveries underscore the critical roles of SCFAs in regulating antibody production, B-cell development, and IgA responses, highlighting their significance in the pathogenesis of immune-related inflammatory conditions, including IBD.

### 3.2 The bifaceted role of bile acid dysmetabolism in IBD

Dysregulation of bile acid metabolism exerts a bifaceted influence on intestinal inflammation in IBD, with primary and secondary bile acids contributing through distinct mechanisms (19). Primary bile acids (e.g., cholic acid, chenodeoxycholic acid) are synthesized in the liver and recycled via enterohepatic circulation. Their metabolic dysregulation is closely linked to IBD: fecal conjugated primary bile acids are significantly increased in IBD patients, while serum secondary bile acids are decreased, alongside impaired deconjugation, transformation, and desulfation processes (20). Farnesoid X receptor (FXR) agonists can significantly reduce pro-inflammatory cytokine secretion from lamina propria mononuclear cells in IBD patients; however, FXR expression and fibroblast growth factor 19 levels are downregulated in intestinal biopsies from CD patients, indicating impaired primary bile acid signaling (21). Additionally, high-fat diet-induced increases in taurine-conjugated bile acids can promote the expansion of bilophilic Bilophila wadsworthia, which produces hydrogen sulfide and can trigger colitis in susceptible hosts.

Secondary bile acids exhibit concentration-dependent “double-edged sword” effects in IBD. On one hand, deoxycholic acid (DCA) and lithocholic acid (LCA) can ameliorate DSS-induced colitis via TGR5 signaling (22), inhibit chemokine expression, and help maintain colonic RORγt^+^ regulatory T-cell homeostasis. Depletion of microbial bile acid metabolism genes leads to a reduction in this protective T-cell subset, while a diverse bile acid pool exerts anti-inflammatory effects through the vitamin D receptor (VDR) axis (23). On the other hand, abnormally elevated levels of secondary bile acids can disrupt the intestinal barrier: high concentrations of DCA reduce tight junction protein expression and transepithelial electrical resistance, and activate the macrophage S1PR2-cathepsin B-NLRP3 inflammasome axis, leading to intestinal epithelial apoptosis (22, 24). Notably, while sulfation of LCA in the colonic epithelium serves as a detoxification mechanism, this modification abrogates its anti-inflammatory properties. The accumulation of secondary bile acids is also associated with an increased risk of colorectal cancer and obesity-related hepatocellular carcinoma, underscoring the critical impact of their concentration and metabolic modifications on IBD outcomes.

### 3.3 The role of tryptophan depletion in IBD pathogenesis

Tryptophan (Trp) metabolism plays a crucial anti-inflammatory role in IBD through the production of microbial aryl hydrocarbon receptor (AhR) ligands. A cohort study involving 535 IBD patients demonstrated an inverse correlation between disease activity and Trp levels, accompanied by activation of the kynurenine pathway, consistent with previous reports (25). Animal studies have shown that gut microbiota from Card9^-/-^ mice exhibit a reduced capacity to activate AhR, associated with decreased levels of indole-3-acetic acid (IAA). Administration of IL-22 alleviated the susceptibility of these mice to DSS-induced colitis, identifying IL-22 as a key mediator of AhR-dependent protection (25). Several microbiota-derived Trp metabolites have demonstrated protective effects: oral administration of *Lactobacillus reuteri*, which produces indole-3-propionic acid (IPA), ameliorated colitis in mice via an IL-10-dependent mechanism, and serum indole levels were significantly reduced in UC patients (26). Similarly, the herbal formula Wu Mei Wan was shown to promote Trp metabolism towards IAA and indole-3-acrylic acid, reinforcing intestinal barrier function and suppressing colonic inflammation in a mouse colitis model via the *Lactobacillus*-IAA-AhR axis (27). Currently, only a limited number of bacteria are known to produce AhR agonists, including *Peptostreptococcus russellii* and members of the genus *Lactobacillus*. The mechanism of IPA production is best characterized in *Clostridium sporogenes*, where it is synthesized via the fldAIBC gene cluster responsible for phenyllactate metabolism. IPA exerts its barrier-protective and anti-inflammatory effects, including inhibition of mucosal tumor necrosis factor production, primarily through activation of the pregnane X receptor (PXR) (28).

## 4 Potential mechanisms of gut microbiota metabolites in IBD therapy

The preceding discussion on the involvement of gut microbial metabolites in IBD pathogenesis suggests that therapeutic modulation of these dysregulated metabolites—either by reducing harmful metabolites at their source or by supplementing beneficial ones—represents a promising avenue for IBD treatment (**Table 1**).

**Table 1:** Main mechanisms of action of gut microbiota metabolites.

Metabolites	Receptor		Mechanism	Physiological/Pathological Effect	Dietary/Microbial Intervention	References
**SCFAs**	-		Participate in the tricarboxylic acid cycle	Provide energy	Increase intake of whole grains, legumes, and vegetables; Supplement with probiotics that produce SCFAs such as *Bifidobacterium* and *Lactobacillus*	(32)
	PAPRγ		Limit oxygen diffusion into the intestinal lumen	Inhibit pathogen growth, maintain intestinal homeostasis		(31)
			Inhibit iNOS expression	Reduce NO production, lower luminal nitrate levels		(33)
	GPR41/GPR109A		Inhibit HDAC and signaling pathways such as NF-κB and MAPK, initiate anti-inflammatory cascade signaling	Regulate M1/M2 macrophage polarization, promote IL-10 secretion and barrier repair		(34)
Secondary bile acids	FXR		Associated with intestinal immune regulation and barrier function; Influence response to anti-α4β7 integrin immunotherapy in IBD patients		Control high-fat diet intake; Consume adequate dietary fiber to promote bile acid excretion; Avoid antibiotic abuse to maintain gut microbiota balance	(35)
	GPBAR1/TGR5					
	VDR		Regulate the Th17/Treg axis balance			(23)
	RORγt					(36)
Tryptophan metabolites	IEt IPyA I3A	AhR	Promote IL-22 secretion	Maintain the integrity of the apical junction complex	Consume adequate high-quality protein (eggs, lean meat, soy products); Supplement with tryptophan-containing foods and probiotics	(37)
		DRD2	Activate the Gβγ-PLC-PKCθ signaling pathway, promote N-WASP degradation	Mediate colonization resistance against enteric pathogens		(38)
	XANA/KYNA	AhR	Promote IL-22 secretion; Promote intestinal epithelial cell survival and proliferation; Promote glycolysis in T cells	Reduce the severity of colitis		(39)
	IAA	AhR/Regulate gut microbiota and their metabolites	Enrich *Bifidobacterium pseudolongum* and its metabolite equol in the colon	Increase the proportion of Foxp3+ T cells in colon tissue, alleviate colitis		(40)
	ILA		Increase the abundance of other tryptophan-metabolizing bacteria, promote the synthesis of other indole derivatives	Alleviate DSS-induced colitis and IL-10-/- spontaneous colitis		(41)
	IPA	PXR	Involved in TLR signaling pathway	Regulate the intestinal stroma, inhibit inflammation and fibrosis		(28)
Lipid metabolites	PUFAs	TLR2	Activate IRE1α in the endoplasmic reticulum of intestinal epithelial cells	Trigger expression of pro-inflammatory factors such as IL-8 and TNF-α	Reduce intake of ω-3 and ω-6 PUFAs abundant in Western diets	(42)
	Linoleic Acid	AhR	Decrease the Th17/Treg ratio	Exert anti-colitis effects	Appropriately increase dietary linoleic acid intake	(43)
	Succinate	SUCNR1	Mediate pro-inflammatory polarization of macrophages and fibroblast activation	Exacerbate intestinal inflammation and fibrosis	Reduce succinate intake	(44)
	Polyamines (Putrescine, Spermidine, Spermine)	K ion channel receptor	Inhibit the assembly of the NLRP3 inflammasome in colon tissue	Maintain intestinal mucosal integrity	Consume fermented foods in moderation (yogurt, kimchi); Avoid excessive intake of red meat; Maintain gut microbiota balance	(45)
Amino acids and derivatives	L-Lysine	Colonic dendritic cells	AhR-IDO1-kynurenine pathway	Balance Th17/Treg immune responses	Repair intestinal mucosal barrier function	(46)
	Phenylalanine	ADRs	Increased production of phenylacetylglutamine (PAGln), inducing colonic DNA damage via the ADR-AMPK signaling pathway; Enhance platelet activation and plasma CD40L expression	Increase susceptibility to colitis	Reduce abundance of phenylacetic acid-producing bacteria (e.g., Proteobacteria)	(47, 48)
	BCAAs: Leucine, Isoleucine, Valine	GPR109	1. Inhibit the TLR4/MyD88/NF-κB signaling pathway, reduce secretion of pro-inflammatory cytokines (e.g., TNF-α, IL-6);	1. Alleviate intestinal inflammation; 2. Reduce intestinal permeability, prevent bacterial and toxin translocation, decrease inflammatory triggers	Consume protein in a balanced manner, avoid excessive BCAA supplementation	(49)
Emerging metabolites	5-HT	5-HT3 receptor and 5-HT transporter	NLRP3-IL-1β inflammatory pathway	Exacerbate DSS-induced colitis	The natural compound Batatasin III shows therapeutic potential in slow-transit constipation models by modulating 5-HT and Substance P levels, improving intestinal motility, and inhibiting the NLRP3-IL-1β inflammatory pathway.	(50, 51)
	Substance P	Tachykinin receptor 1 (TACR1)	Downregulate immunomodulatory molecules such as LAP1, PD-L1, CD73; Promote effector CD4 T cell proliferation	Induce intestinal mucosal inflammation		(52)

SCFAs, short-chain fatty acids; PPARγ, peroxisome proliferator-activated receptor gamma; GPR, G protein-coupled receptor; TLR, Toll-like receptor; iNOS, inducible nitric oxide synthase; HDAC, histone deacetylase; NF-κB, nuclear factor-kappa B; MAPK, mitogen-activated protein kinase; NLRP3, NOD-like receptor family pyrin domain containing 3; NO, nitric oxide; IL, interleukin; FXR, farnesoid X receptor; GPBAR1/TGR5, G protein-coupled bile acid receptor 1; VDR, vitamin D receptor; RORγt, retinoic acid receptor-related orphan receptor gamma t; IBD, inflammatory bowel disease; Treg, regulatory T cell; ILC, innate lymphoid cell; IEt, indole-3-ethanol; IPyA, indole-3-pyruvic acid; I3A, indole-3-aldehyde; XANA, xanthurenic acid; KYNA, kynurenic acid; IAA, indole-3-acetic acid; ILA, indole-3-lactic acid; IPA, indole-3-propionic acid; AhR, aryl hydrocarbon receptor; DRD2, dopamine receptor D2; PXR, pregnane X receptor; DSS, dextran sulfate sodium; PUFAs, polyunsaturated fatty acids; LA, linoleic acid; Lys, L-lysine; SUCNR1, succinate receptor 1; IRE1α, inositol-requiring enzyme 1 alpha; STAT, signal transducer and activator of transcription; CD, Crohn’s disease; TNF, tumor necrosis factor; “-” indicates not mentioned in the text and not detailed in the references cited; BCAAs, Branched-chain amino acids; 5-HT, 5-Hydroxytryptamine. TACR1, Tachykinin receptor 1.

### 4.1 Potential mechanisms of SCFAs in IBD therapy

The intestinal lumen harbors the highest concentrations of SCFAs, where their anti-inflammatory and immunomodulatory properties counteract inflammatory cascades triggered by “leaky gut” associated with various intestinal disorders. This section synthesizes research progress on SCFAs in IBD, systematically delineating their multifaceted mechanisms of action ranging from energy metabolism to complex immune regulation.

Metabolic foundation and maintenance of intestinal homeostasis by SCFAs (29). SCFAs are primarily produced by bacteria from the phyla *Bacteroidetes* (acetate, propionate) and *Firmicutes* (butyrate) (30). They serve not only as crucial energy substrates for colonic epithelial cells (primarily utilizing butyrate) and peripheral tissues (primarily utilizing acetate and propionate) but also as key regulators of intestinal homeostasis (31). Their homeostatic mechanisms include: 1) Providing energy through β-oxidation in colonocytes and activating peroxisome proliferator-activated receptor gamma (PPARγ), thereby limiting oxygen diffusion into the lumen and maintaining an anaerobic environment (32, 33); 2) Inhibiting inducible nitric oxide synthase (iNOS) expression, reducing nitric oxide (NO) and subsequent nitrate production, which curbs the overgrowth of potentially pathogenic bacteria such as Enterobacteriaceae and secures the dominance of beneficial obligate anaerobes (32, 33). These functions collectively form the metabolic foundation for the anti-inflammatory effects of SCFAs.

The core immunomodulatory effects of SCFAs are primarily mediated through the activation of G protein-coupled receptors (GPCRs), such as free fatty acid receptors 2/3 (FFAR2/3, i.e., GPR43/41) and hydroxycarboxylic acid receptor 2 (HCA2, i.e., GPR109A), as well as through inhibition of histone deacetylases (HDACs) (29). These mechanisms act synergistically to suppress inflammation and promote repair at multiple levels (**Figure 1B**).

#### 4.1.1 Regulation of innate immunity and cytokine networks

SCFAs broadly regulate intestinal inflammation and immune homeostasis by acting on various innate immune cells, including macrophages, dendritic cells (DCs), innate lymphoid cells (ILCs), and neutrophils. In macrophages, butyrate downregulates the pro-inflammatory cytokines tumor necrosis factor-alpha (TNF-α) and interleukin-6 (IL-6) while upregulating the anti−inflammatory cytokine IL-10 through the inhibition of histone deacetylases (HDACs) and the nuclear factor−kappa B (NF−κB) pathway (29). Propionate suppresses M1 macrophage polarization and promotes a reparative M2 phenotype by modulating the mitogen−activated protein kinase (MAPK) pathway, including p38, ERK, and JNK (34). Additionally, butyrate activates the G protein−coupled receptor GPR109A, which enhances macrophage phagocytosis and inhibits NLRP3 inflammasome activation (53). In dendritic cells, SCFAs (particularly butyrate and propionate) reduce the expression of co−stimulatory molecules CD80/CD86 and pro−inflammatory cytokines (e.g., IL−12, IL−23) via HDAC inhibition, thereby limiting the differentiation of Th1 and Th17 cells (15). Concomitantly, SCFAs promote the production of retinoic acid and TGF−β by DCs, favoring the induction of regulatory T cells (Tregs). Regarding innate lymphoid cells and neutrophils, butyrate suppresses the production of IFN−γ and IL−17 by ILC1 and ILC3 while enhancing IL−22 production by ILC3 through GPR41 and GPR43 signaling, thus reinforcing epithelial barrier repair (54, 55). Furthermore, SCFAs reduce neutrophil recruitment to sites of inflammation and inhibit the release of elastase and reactive oxygen species (ROS), thereby attenuating tissue damage (56).

At the level of signaling pathways and molecular targets, SCFAs primarily modulate the NF−κB, MAPK (p38/ERK/JNK), JAK−STAT, and PI3K/Akt pathways (57–59). The involved receptors include GPR41, GPR43, and GPR109A; key enzymes and proteins include HDACs (notably HDAC1/2/3) and NLRP3; and the relevant effector molecules include TNF−α, IL−6, IL−1β, IL−10, IL−22, IFN−γ, ROS, and NO (60). In summary, SCFAs exert anti−inflammatory and tissue−protective effects in IBD through synergistic actions across multiple cell types, signaling pathways, and molecular targets, thereby finely regulating innate immune responses and cytokine networks (61).

#### 4.1.2 Enhancement of epithelial barrier function

SCFAs reinforce the intestinal epithelial barrier through multiple coordinated mechanisms. They signal via epithelial GPCRs, and inhibit HDACs, leading to the activation of transcription factors including hypoxia-inducible factor-1 (HIF-1) and signal transducer and activator of transcription 3 (STAT3). These events enhance the expression of tight junction proteins (e.g., claudin, occludin, ZO-1), promote the production of antimicrobial peptides (such as β-defensins), and stimulate epithelial cell proliferation, thereby strengthening the physical barrier (29). Additionally, SCFAs can downregulate myosin light chain kinase (MLCK) activity, reducing paracellular permeability, and inhibit epithelial cell apoptosis, further preserving barrier integrity (62).

Experimental studies have provided direct evidence for these barrier-protective effects. Oral administration of butyrate has been shown to restore goblet cell number and function in DSS-induced colitis mice by promoting M2 macrophage polarization via the Wnt-ERK1/2 axis, thereby repairing the mucus barrier (62). Propionate acts through GPR41 to promote goblet cell differentiation and upregulate mucin gene expression (e.g., *Muc2*), contributing to the mucus layer’s integrity (63, 64). Furthermore, acetate has been reported to enhance tight junction assembly via GPR43-mediated activation of AMPK (53). Collectively, SCFAs exert multifaceted protection of the epithelial barrier, encompassing both the physical (tight junction) and chemical (mucus and antimicrobial peptides) components (65), which is critical for counteracting the “leaky gut” phenomenon in IBD.

#### 4.1.3 Regulation of adaptive immune balance

SCFAs are crucial for maintaining immune equilibrium. They promote the production of the epithelial repair cytokine IL-22 by CD4+ T cells and innate lymphoid cells (66). More importantly, SCFAs, particularly butyrate, significantly induce the differentiation of naïve T cells into regulatory T cells (Tregs) by promoting histone H3 acetylation at the Foxp3 locus, while inhibiting the development of pro-inflammatory T helper 17 (Th17) cells, thereby correcting the Th17/Treg imbalance central to IBD pathogenesis (67–69). Furthermore, SCFAs can induce the secretion of the repair cytokine IL-18 by activating Toll-like receptors (TLRs) and the NOD-like receptor protein 3 (NLRP3) inflammasome, modulating local immune homeostasis (70–72).

Despite substantial evidence supporting the therapeutic potential of SCFAs (especially butyrate), studies also indicate that under specific contexts (e.g., severe barrier disruption, acute T-cell-mediated inflammation), high concentrations of SCFAs may exacerbate inflammation and tissue damage by metabolically reprogramming CD4+ T cells and enhancing their effector functions (73). This paradoxical pro-inflammatory effect underscores the complexity and context-dependency of SCFA actions.

In summary, SCFAs constitute a multi-target anti-inflammatory and reparative network by providing energy, maintaining an anaerobic environment, modulating diverse immune cells, and strengthening the epithelial barrier. Current research robustly supports the substantial promise of SCFA supplementation—either through direct administration of SCFAs themselves or via probiotics that produce them—for IBD therapy.

### 4.2 Potential mechanisms of bile acids in IBD therapy

Bile acids are amphipathic molecules synthesized from cholesterol in the liver through a series of enzymatic reactions, playing a critical role in the digestion and absorption of lipids. The primary bile acids synthesized in humans are cholic acid (CA) and chenodeoxycholic acid (CDCA). They are conjugated with glycine or taurine, secreted into bile, and released into the small intestine postprandially. Approximately 95% of bile acids are efficiently reabsorbed in the terminal ileum and returned to the liver via the portal vein, completing the enterohepatic circulation. The remaining fraction enters the colon, where it undergoes extensive biotransformation by the gut microbiota: bile salt hydrolases (BSH) deconjugate them to yield free bile acids; subsequently, bacterial 7α-dehydroxylases convert CA and CDCA into the secondary bile acids deoxycholic acid (DCA) and lithocholic acid (LCA), respectively. The microbiota can also generate other active molecules, such as ursodeoxycholic acid (UDCA) and various oxo-bile acid analogs, through reactions like epimerization (74, 75).

Compared to healthy individuals, IBD patients exhibit reduced abundance of genes from bile acid-transforming bacteria (e.g., *Firmicutes*) in the gut, leading to significantly decreased levels of secondary bile acids (e.g., DCA, LCA) and correspondingly elevated levels of primary bile acids (76). This “bile acid dysmetabolism” is considered a critical factor in IBD pathogenesis, although its specific mechanisms are complex and bidirectional (7).

#### 4.2.1 Bile acids exert broad physiological functions by activating multiple receptors

The farnesoid X receptor (FXR) and the GPBAR1/TGR5 are central regulators. FXR is crucial for maintaining intestinal barrier integrity. FXR-deficient mice exhibit gut dysbiosis, increased intestinal permeability, and bacterial translocation (77). In experimental colitis models (DSS or TNBS-induced), the FXR agonist obeticholic acid (INT-747) effectively attenuates inflammation, protects the epithelial barrier, and reduces pro-inflammatory cytokine production (78). Similarly, TGR5 exerts protective effects. TGR5 knockout mice display impaired intestinal mucosal barriers and heightened susceptibility to TNBS-induced colitis (79). Mechanistically, TGR5 deficiency impairs IL-10 signaling and reduces the generation of regulatory T cells (Tregs) and M2 macrophages, whereas the TGR5 agonist BAR501 prevents colitis in an IL-10-dependent manner (79). Additionally, bile acids participate in immune modulation through other nuclear receptors such as the vitamin D receptor (VDR) (23) (**Figure 1C**).

#### 4.2.2 Bile acids directly maintain and repair the intestinal epithelial barrier

Bile acids can promote intestinal stem cell renewal and epithelial regeneration via TGR5 activation (80). UDCA and its metabolite LCA have been shown to inhibit the release of pro-inflammatory cytokines like TNF-α and IL-6, reduce epithelial cell apoptosis, thereby promoting mucosal healing and attenuating DSS-induced inflammation (81). However, specific bile acids can also impair the barrier under certain conditions. For instance, excessive DCA resulting from a high-fat diet can hinder epithelial healing by activating FXR and suppressing cystic fibrosis transmembrane conductance regulator (CFTR) and fibroblast growth factor (FGF) signaling (82). DCA can also activate the sphingosine-1-phosphate receptor 2 (S1PR2)-ERK signaling pathway, triggering cathepsin B release and NLRP3 inflammasome activation, thereby exacerbating inflammation (82). Conversely, the secondary bile acid LCA can inhibit NLRP3-mediated inflammation by promoting its ubiquitination and degradation via the TGR5-cAMP-PKA axis (83). This highlights the highly species- and context-dependent nature of bile acid effects.

Regarding immune regulation, bile acids, particularly secondary bile acids and their derivatives, are key modulators of the Th17/Treg cell balance. A derivative of LCA, 3-oxolithocholic acid (3-oxoLCA), directly binds to and inhibits RORγt, the key transcription factor for Th17 cells, thereby suppressing their differentiation. Another derivative, isoallolithocholic acid (isoalloLCA), promotes Treg cell differentiation and upregulates Foxp3 expression by inducing mitochondrial reactive oxygen species (mitoROS) production (36). Administration of these two compounds in animal models reduces intestinal Th17 cells, increases Treg cells, and alleviates colitis (84). Bile acids also maintain the activity of RORγ+ Treg cells via VDR (85) and inhibit NF-κB-dependent pro-inflammatory cytokine expression in macrophages by activating TGR5 (86). In preclinical studies, UDCA supplementation attenuated colitis and downregulated the expression of mucosal addressin cell adhesion molecule-1 (MAdCAM-1) (85).

In recent years, the relevance of bile acid metabolism to the efficacy of IBD therapies has gained increasing attention. Studies indicate that the gut microbiota influences patient responses to biologics such as anti-α4β7 integrin by modulating bile acid metabolism. Patients or model mice in remission show increased abundance of beneficial bacteria like Lactobacillus and Clostridium in the gut, positively correlating with elevated levels of secondary bile acids such as LCA and DCA, suggesting a potential mechanism for therapeutic efficacy (87, 88). Interestingly, elevated levels of secondary bile acids like LCA and DCA in the gut of cholecystectomized patients and model mice were found to inhibit monocyte/macrophage recruitment to the intestine and reduce inflammation (89), hinting at their potential anti-inflammatory role. However, the role of DCA remains controversial, as other studies indicate it can induce GSDMD-mediated pyroptosis and IL-1β release in macrophages (90), or trigger ferroptosis in intestinal epithelial cells, exacerbating intestinal injury (91).

In conclusion, bile acids, as key signaling molecules within the intestinal microenvironment, are deeply involved in maintaining the intestinal barrier, regulating immune homeostasis, and modulating inflammatory processes in IBD through a complex network of receptors and metabolic pathways. They can act as protective factors or, when dysregulated, contribute to tissue damage. Elucidating the precise roles of individual bile acid species in specific pathological contexts, and developing therapeutic strategies targeting bile acid metabolic axes (e.g., FXR/TGR5), holds significant promise for the discovery of novel diagnostic biomarkers and treatments for IBD.

### 4.3 Potential mechanisms of tryptophan metabolites in IBD therapy

Tryptophan (Trp), an essential aromatic amino acid obtained exclusively from the diet, serves as a critical precursor for numerous bioactive molecules. Its metabolism in the body proceeds via three principal pathways: the host-driven kynurenine (KYN) and serotonin (5-HT) pathways, and the microbiota-driven indole pathway. Approximately 95% of dietary Trp is catabolized via the KYN pathway, 1%-2% is converted to 5-HT and melatonin, while the remaining 4%-6% is metabolized by the gut microbiota into various indole derivatives (35). These metabolites constitute a complex signaling network that profoundly influences intestinal immune homeostasis, barrier function, and inflammatory responses by activating specific receptors—including the aryl hydrocarbon receptor (AhR), pregnane X receptor (PXR), and dopamine receptor D2 (DRD2)—thereby linking tightly to the pathogenesis, progression, and prognosis of IBD. Notably, levels of anti-inflammatory microbiota-derived Trp metabolites, such as indole-3-propionic acid (IPA) and indole-3-acetic acid (IAA), are significantly reduced in the feces of IBD patients (**Figure 1D**). Receptors including AhR, PXR, and DRD2 represent common nodes through which these metabolites exert their effects. A hallmark of IBD is the systemic dysregulation of Trp metabolic pathways, characterized by a reduction in anti-inflammatory metabolites (e.g., KYNA, IPA) and a relative increase in pro-inflammatory metabolites (e.g., quinolinic acid), accompanied by attenuated signaling through their cognate receptors. Consequently, strategies aimed at reprogramming this aberrant Trp metabolism—through dietary interventions, modulation with probiotics/prebiotics, direct supplementation with key metabolites (e.g., IPA, KYNA), or even synthetic biology approaches (e.g., engineered bacteria expressing AADAT)—have emerged as highly promising therapeutic avenues for IBD.

#### 4.3.1 The kynurenine pathway as a central immunomodulatory axis

This pathway is initiated by indoleamine 2,3-dioxygenase-1 (IDO-1), which is highly expressed in intestinal epithelial and lamina propria cells during active IBD (92). IDO-1 activation induces immune tolerance and promotes the production of anti-inflammatory cytokines IL-10 and TGF-β. Its expression can be induced by probiotics such as Bifidobacterium, thereby ameliorating colitis and increasing regulatory T cell (Treg) populations (93). Metabolites derived from this pathway exhibit dual immunomodulatory functions. Kynurenic acid (KYNA) and xanthurenic acid (XANA) are primary anti-inflammatory mediators that maintain epithelial barrier integrity and inhibit pro-inflammatory cytokine release by activating G protein-coupled receptor GPR35 or AhR. Clinical studies have revealed an inverse correlation between serum KYNA levels and inflammatory severity in IBD patients, and supplementation with KYNA or XANA attenuates experimental colitis (25, 39, 94). Conversely, quinolinic acid exerts pro-inflammatory and neurotoxic effects. Modulating this metabolic balance has recently emerged as a therapeutic strategy; for instance, exogenous administration of the recombinant enzyme AADAT directs substrate flux towards KYNA and XANA synthesis while inhibiting quinolinic acid production, thereby exerting protective effects in colitis models (39).

#### 4.3.2 The microbial indole pathway: A bridge between microbiota and host immunity

Gut bacteria, including Clostridium and Lactobacillus species, convert Trp into bioactive molecules such as indole-3-propionic acid (IPA), indole-3-lactic acid (ILA), and indole-3-acetic acid (IAA) (41). These indole derivatives serve as high-affinity ligands for AhR and PXR. By activating AhR, they promote IL-22 secretion from intestinal epithelial cells, which is crucial for barrier repair and host defense. Deficiency of the IBD susceptibility gene CARD9 impairs the capacity of the gut microbiota to produce AhR ligands, thereby exacerbating colitis (37, 95). Concurrently, IPA, as a PXR ligand, directly targets intestinal epithelial cells to downregulate inflammatory cytokines like TNF-α and enhance tight junction protein expression, thus preserving barrier function (28). Importantly, IPA and its interaction with PXR have been shown to significantly inhibit intestinal fibrosis—a key pathological process underlying stricture formation in CD and wall stiffening in UC. Clinical data indicate reduced fecal IPA levels and decreased intestinal PXR expression in active IBD patients, correlating with increased expression of fibrosis-related genes; supplementation with IPA specifically attenuates colonic fibrosis in murine models (96). Furthermore, L-tryptophan derivatives, such as indole-3-pyruvic acid (IPyA), indole-3-propionic acid (IPA), and indole-3-ethanol, are produced through gut microbiota metabolism and can activate the neurotransmitter receptor dopamine receptor D2 (DRD2) in the intestinal epithelium, thereby protecting the host against enteric pathogens (38) (**Figure 1D**).

#### 4.3.3 The serotonin pathway: A complex mediator in neuro-immune crosstalk

Serotonin (5-HT) is primarily synthesized by enterochromaffin cells in the gut. In IBD patients, levels of its synthetic enzymes and transporters are frequently dysregulated, and its utilization is increased (97). 5-HT exerts dual effects by binding to various specific receptors (5-HTR1, 3, 4, 7, *etc.*) on immune cells such as dendritic cells and macrophages: it can both promote macrophage polarization towards an anti-inflammatory phenotype and stimulate the production of pro-inflammatory cytokines including IL-6, TNF-α, and IL-8 (98). Additionally, 5-HT itself can activate AhR, contributing to immunomodulation (**Figure 1D**). It also serves as a critical signaling molecule within the enteric nervous system, indirectly regulating intestinal inflammation through neuro-immune interactions.

IBD, inflammatory bowel disease. SCFA, short-chain fatty acids. BAs, bile acids. SBAs, secondary bile acids. DCA, Deoxycholic acid. LCA, Lithocholic acid. NF-κB, Nuclear factor kappa-light-chain-enhancer of activated B cells. TNF-α, Tumor necrosis factor-alpha. IL-6, Interleukin-6. IPA, Indole-3-propionic acid. IAA, Indole-3-acetic acid. MAPK, Mitogen-activated protein kinase. FXR, farmesoid X receptor; GPR, G-protein coupled receptor. XANA, xanthurenic acid. KYNA, kynurenic acid. 5-HT, 5-hydroxytryptamine.

### 4.4 Potential mechanisms of other metabolites in IBD therapy

Beyond the well-characterized roles of SCFAs, bile acids, and tryptophan metabolites, numerous other gut-derived metabolites exhibit significant alterations in IBD and profoundly influence disease progression and immune homeostasis. These metabolites originate from diverse sources, including dietary components, host-microbe co-metabolism, and intermediates of energy metabolism, and their mechanisms of action are often complex and context-dependent.

#### 4.4.1 Lipid metabolites

Dietary polyunsaturated fatty acids (PUFAs) are closely linked to IBD risk, although their effects remain controversial. For instance, ω-3 and ω-6 PUFAs, abundant in Western diets, can be recognized by toll-like receptor 2 (TLR2) via oxidation-specific epitopes, activating the endoplasmic reticulum stress sensor IRE1α in intestinal epithelial cells. In CD patients with impaired glutathione peroxidase 4 expression, this triggers the expression of pro-inflammatory cytokines such as IL-8 and TNF-α (42). However, another study suggests that microbiota-derived linoleic acid (a PUFA) can act as an aryl hydrocarbon receptor (AhR) ligand, suppressing Th17 cell differentiation while promoting STAT1-mediated regulatory T cell (Treg) differentiation, thereby reducing the Th17/Treg ratio and exerting anti-colitis effects (43). These disparate findings likely reflect differences in dosage, exposure duration (acute vs. chronic), and the specific microbial context.

**Figure 2:**
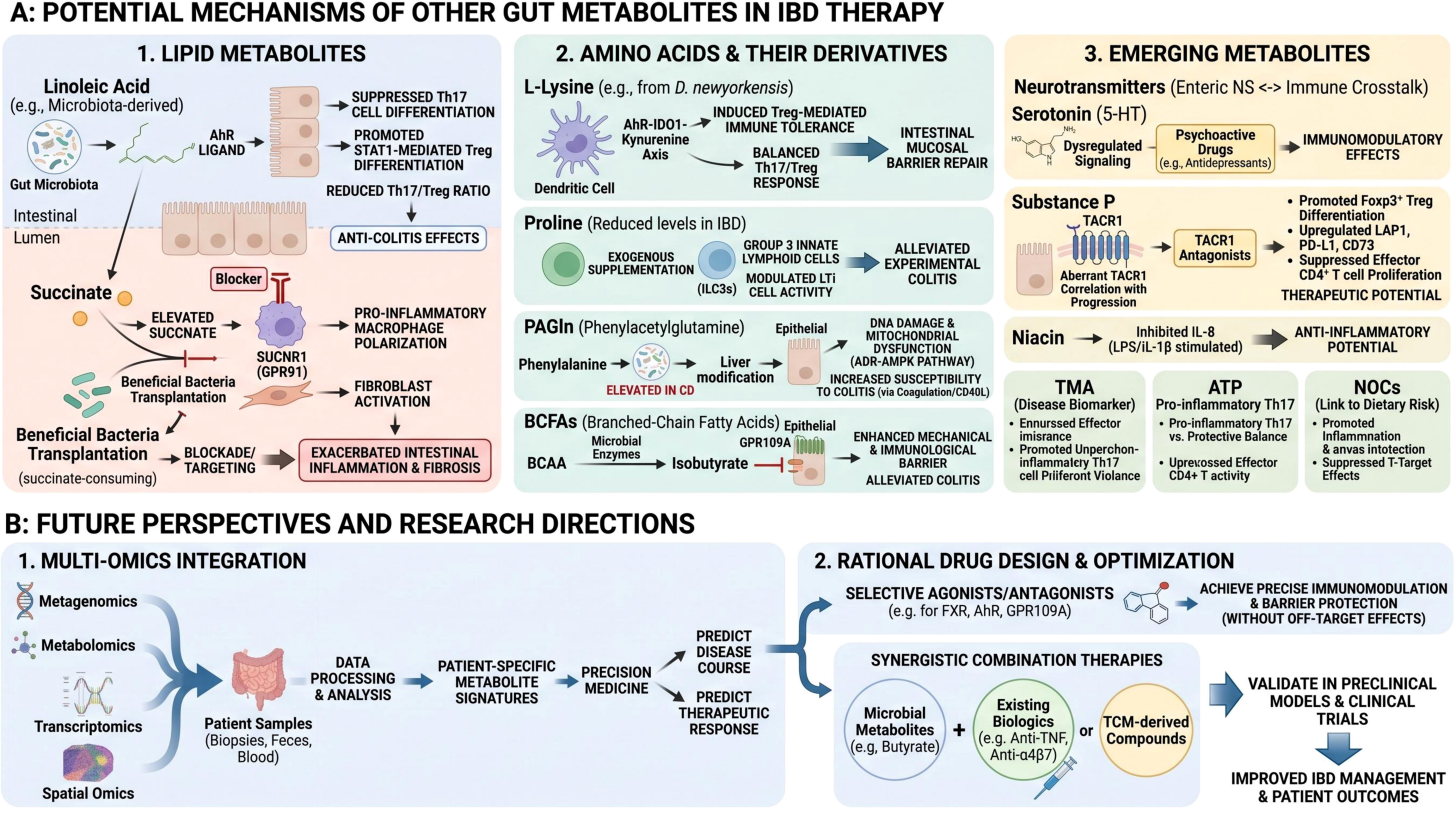
Potential mechanisms of gut microbial metabolites in inflammatory bowel disease therapy and future research directions. **(A)** Overview of how gut-derived metabolites, other than the well-known short-chain fatty acids, may influence inflammatory bowel disease (IBD) treatment. Some of these metabolites appear to help control inflammation and protect the intestinal mucosal barrier. For example, (1) lipid metabolites such as linoleic acid may act through aryl hydrocarbon receptor (AhR) signaling. In this way, they can limit Th17 cell differentiation while supporting regulatory T cell (Treg) responses, which may help reduce intestinal inflammation. Succinate, however, seems to have a more harmful role under certain conditions. By activating SUCNR1/GPR91 signaling, it may promote pro-inflammatory macrophage polarization and fibroblast activation, potentially worsening intestinal inflammation and fibrosis. (2) Amino acids and their derivatives are also involved in this process. Molecules such as lysine, proline, phenylacetylglutamine (PAGln), and branched-chain fatty acids (BCFAs) may affect mucosal repair and immune balance through pathways such as the AhR–IDO1–kynurenine axis, ILC3 activity, epithelial mitochondrial function, and GPR109A signaling. These mechanisms suggest that amino acid-related metabolism could be closely linked to both barrier protection and inflammatory regulation in IBD. (3) Emerging metabolites, including neurotransmitters, substance P, niacin, trimethylamine (TMA), ATP, and N-nitroso compounds (NOCs). These molecules may participate in communication between the enteric nervous system and the immune system, influence T-cell activity, and contribute to disease progression. **(B)** Looking ahead, a better understanding of these metabolite-driven mechanisms will likely depend on integrating multiple omics approaches, such as metagenomics, metabolomics, transcriptomics, and spatial omics. Such strategies may help identify metabolite signatures that are specific to individual patients. They could also guide more precise therapeutic approaches, including receptor-targeted agonists or antagonists, combinations of microbial metabolites with biologics, and therapies based on traditional Chinese medicine-derived compounds.

Succinate, a tricarboxylic acid cycle intermediate and microbial fermentation product, is elevated in the serum and intestinal lumen of CD patients, with increased expression of its receptor, SUCNR1, in intestinal tissue and fibroblasts. SUCNR1 activation mediates pro-inflammatory macrophage polarization and fibroblast activation, exacerbating intestinal inflammation and fibrosis (44). Targeting the succinate-SUCNR1 axis—for example, through receptor blockade or transplantation of succinate-consuming beneficial bacteria—represents a potential therapeutic strategy (99). Among the polyamines—putrescine, spermidine, and spermine—spermine has been shown to inhibit NLRP3 inflammasome assembly by modulating intracellular potassium flux, thereby suppressing macrophage pyroptosis. Furthermore, administration of the AhR pro-ligand indole-3-carbinol (I3C) combined with spermine significantly alleviated pathological symptoms in a murine colitis model (45) (**Figure 2A**).

#### 4.4.2 Amino acids and their derivatives

Beyond tryptophan, metabolism of other amino acids is extensively involved in IBD immunomodulation. Studies have shown significantly reduced fecal proline levels in IBD patients. Exogenous proline supplementation alleviates experimental colitis by activating group 3 innate lymphoid cells (ILC3s) and modulating lymphoid tissue inducer cell activity (100). L-lysine, derived from specific commensal bacteria such as Dubosiella newyorkensis or Clostridium innocuum, acts on colonic dendritic cells to activate the AhR-IDO1-kynurenine axis. This induces Treg-mediated immune tolerance, balances the Th17/Treg response, and promotes intestinal mucosal barrier repair (46).

Conversely, phenylacetylglutamine (PAGln), a co-metabolite generated from phenylalanine by the gut microbiota and further modified by the liver, exerts detrimental effects. PAGln induces colonic DNA damage and mitochondrial dysfunction via the ADR-AMPK signaling pathway (47), and enhances platelet activation and plasma CD40L expression, upregulating coagulation-related pathways and increasing susceptibility to colitis (48). Notably, PAGln levels are significantly elevated in CD patients, an alteration closely associated with high-protein diets and increased abundance of phenylacetic acid-producing bacteria, such as *Proteobacteria* (48).

Branched-chain amino acids (BCAAs)—leucine, isoleucine, and valine—can be converted by microbial enzymes into BCFAs, including isobutyrate, isovalerate, and 2-methylbutyrate. Although BCFAs constitute a minor fraction of total intestinal SCFAs (with straight-chain fatty acids like acetate and butyrate present at concentrations of ~20–140 mmol/L), their concentration increases from the proximal to the distal colon (averaging 4.6 mmol/L in the proximal colon and 6.3 mmol/L in the distal colon), with a total daily systemic production of approximately 11.1 mmol, suggesting significant physiological relevance both locally and systemically (49). Among BCFAs, isobutyrate has been shown to activate G protein-coupled receptor 109A (GPR109A), inhibiting the TLR4/MyD88/NF-κB signaling pathway and enhancing both the mechanical and immunological functions of the colonic barrier, thereby alleviating colitis (49) (**Figure 2A**).

#### 4.4.3 Emerging metabolites

Gut-derived neurotransmitters, key mediators of crosstalk between the enteric nervous system and the immune system, have emerged as a frontier in IBD therapeutic research (101). Serotonin (5-HT), produced by enterochromaffin cells, is one of the most extensively studied gut-derived neurotransmitters. Recent reviews indicate that dysregulation of 5-HT signaling is closely linked to IBD pathogenesis (102). Interventions targeting this pathway, including psychoactive drugs such as antidepressants and hallucinogens, may exert immunomodulatory effects by modulating 5-HT signaling, offering new avenues for combination therapies in IBD (50). Substance P, a member of the tachykinin family, participates in neuroimmune communication by binding to tachykinin receptor 1 (TACR1). Aberrant TACR1 expression in IBD patients correlates with disease progression (52). TACR1 antagonists have been shown to effectively control intestinal mucosal inflammation by promoting Foxp3^+^ regulatory T cell differentiation, upregulating immunomodulatory molecules such as LAP1, PD-L1, and CD73, and suppressing effector CD4+ T cell proliferation (52). This provides direct evidence supporting the therapeutic potential of targeting the tachykinin signaling pathway in IBD. Furthermore, the natural compound Batatasin III has demonstrated therapeutic potential in a slow-transit constipation model by modulating 5-HT and substance P levels, improving intestinal motility, and inhibiting the NLRP3-IL-1β inflammatory pathway (103).

Niacin (nicotinic acid) has been shown to significantly inhibit LPS- and IL-1β-stimulated IL-8 production *in vitro*, suggesting anti-inflammatory potential and possible utility as a therapeutic target (104). Trimethylamine (TMA) levels are reduced in IBD patients, suggesting its potential as a disease biomarker, although the underlying mechanisms remain to be elucidated (105). ATP, present in the intestinal lamina propria, can exert pro-inflammatory effects by inducing a cytokine milieu that favors Th17 differentiation; however, under physiological conditions, it also plays a protective role in maintaining microbial balance (106). Additionally, high-protein diets promote microbial production of N-nitroso compounds (NOCs), which are significantly elevated in the feces of patients with IBD and colorectal cancer. This may mechanistically link dietary risk factors to disease pathogenesis, highlighting their potential as disease indicators or risk predictors (107) (**Figure 2A**).

## 5 Conclusions and future perspectives

Gut microbial metabolites—particularly SCFAs, bile acids, and tryptophan derivatives—are fundamental determinants of intestinal barrier integrity and key regulators of IBD progression. Through complex interactions with the gut microbiota, intestinal epithelial cells, and innate/adaptive immune cells, these metabolites orchestrate mucosal immune homeostasis and shape the intestinal microenvironment. Therapeutic strategies targeting the gut microbiota and its metabolome, including dietary interventions, probiotics, and bioactive compounds from traditional Chinese medicine (TCM) (108–111), exert synergistic effects by remodeling microbial metabolic profiles, modulating immune balance, enhancing epithelial barrier function, and suppressing local inflammation.

Looking ahead, the integration of multi-omics technologies (transcriptomics, proteomics, metabolomics, spatial omics) will enable systematic identification of functional microbial metabolite signatures in IBD. To advance the field, the following specific research directions are proposed: (1) Integrate metagenomics, metabolomics, and spatial transcriptomics to define patient−specific metabolite signatures that predict disease course and therapeutic response, enabling personalized IBD management. (2) Design and optimize selective agonists/antagonists for key metabolite−sensing receptors (e.g., FXR, TGR5, AhR, PXR, DRD2) to achieve precise immunomodulation and barrier protection without off−target effects. (3) Evaluate synergistic efficacy of combining microbial metabolites (e.g., IPA, butyrate, secondary bile acids) with existing biologics (e.g., anti−TNF, anti−α4β7 integrin) or TCM−derived compounds, and validate in preclinical models and clinical trials (**Figure 2B**).
